# HBV and the Microbiome—PubMed Database Literature Review

**DOI:** 10.3390/idr18030038

**Published:** 2026-04-22

**Authors:** Anna Marija Prince, Indra Zeltiņa, Aigars Reinis, Olga Valciņa, Angelika Krūmiņa

**Affiliations:** 1 Department of Residency (Infectious Diseases), Riga Stradins University, LV-1007 Riga, Latvia; 2 Department of Infectology, Riga Stradins University, LV-1007 Riga, Latvia; 3 Department of Infectology, Riga East Clinical University Hospital, LV-1007 Riga, Latvia; 4 Department of Microbiology, Riga Stradins University, LV-1007 Riga, Latvia; 5 The Institute of Food Safety, Animal Health and Environment BIOR, LV-1007 Riga, Latvia

**Keywords:** hepatitis B, HBV, microbiome, PubMed

## Abstract

Objective: Hepatitis B virus (HBV) is a globally distributed infectious disease affecting the liver. This literature review aims to summarize all available relevant information on the PubMed database about HBV’s connection to the microbiome and to consider possible treatment adjuncts. Materials and methods: Database used: PubMed. Keywords used: “HBV”, “Hepatitis B”, “microbiome”. In the PubMed database, 179 research publications were identified using these keywords; 69 studies were excluded as they were irrelevant or retracted. Of the remaining, 110 were analyzed in this literature review, and four additional literature sources were used to supply background information and context. Information was summarized. The analysed studies in total included 14,814 participants (excluding animal studies), of whom 8564 were HBV-infected individuals. Results: Results characterizing abundance or decrease in specific bacterial, viral, and fungal species are heterogeneous; multiple studies support that the HBV patient oral and fecal microbiome is different from that in healthy controls (HCs) and varies throughout disease progression. The HBV seems to transform the microbiome negatively, leading to dysbiosis and decreased microbial diversity in most studies. Evidence links HBV microbiome changes with influence on HbeAg seroconversion, HBV-DNA load, metabolic pathways, liver cirrhosis, and hepatocellular carcinoma. The research proposes that members of microbiota could potentially promote or protect against liver injury in HBV. Four studies proposed that the plasma virome in HBV patients was primarily composed of members of the *Anelloviridae*. One study researched a parasite (*Entamoeba gingivalis*) in HBV patients. Two studies analyzed HBV patients’ fungal profiles. Conclusions: Microbiota research, although promising, at the present moment is heterogeneous. HBV patients’ microbiota is distinguishable from HCs, and multiple studies have tried to identify the HBV characteristic microbiome; however, more precise information is needed to draw conclusions. Fecal microbiota transplantation and probiotics have the potential to be therapy adjuncts for HBV patients, but more research is needed.

## 1. Introduction

Hepatitis B virus (HBV) is a globally distributed infectious disease affecting the liver. The WHO estimates that more than 254 million people were living with chronic hepatitis B infection in 2022, with 1.2 million new infections each year [1]. In 2022, HBV resulted in an estimated 1.1 million deaths, mostly from cirrhosis and hepatocellular carcinoma [1]. This literature review aims to find available information about HBV’s connection to the microbiome and to consider possible treatment directions.

## 2. HBV Microbiome Function

The microbiome is a functionally active organic entity that has been linked to the formation of different diseases and conditions; however, it can also potentially have a protective role. Overall results characterizing abundance or decrease in specific bacterial, viral, and fungal species are quite heterogeneous; however, evidence supports that the HBV patient oral and fecal microbiome is different from that in the HC and varies throughout disease progression [2,3,4,5,6,7,8,9,10,11,12,13]. Most studies included support lower microbial diversity in HBV patients; however, two studies propose higher diversity or more pronounced microbial richness in HBV patients compared to HCs [12,14]. Two studies found no difference comparing the HBV microbiome to HC ([15] (oral microbiome); [16] fecal microbiome).

Growing evidence supports that in multiple HBV-associated liver conditions, microbiota affect different substance metabolic mechanisms and therefore metabolites [5,14,17,18,19,20,21]. Nevertheless, gut microbiota metabolites without a causal effect on chronic HBV were identified in one publication [22]. In HBV, the intestinal microbial community may act as an independent organ to regulate the body’s metabolic balance, which may affect the organism and further disease progression.

In chronic HBV (CHB) patients, when compared to healthy individuals, analysis of gut microbiota reveals that lipopolysaccharide, glycan biosynthesis, lipid, and butanoate metabolism are significantly elevated [5,23]. The host–microbiota–metabolite interplay, amino acid metabolism, nicotinate and nicotinamide metabolism, starch and sucrose metabolism, steroid biosynthesis, and vitamin metabolism were found to be significantly lower in CHB patients than in HCs [24]. The fecal microbiota of the HBV-liver cirrhosis (HBV-LC) patients show enrichment in the metabolism of glutathione, gluconeogenesis, branched-chain amino acids, nitrogen, and lipids, whereas there was a decrease in the level of aromatic amino acids, bile acids, and cell cycle-related metabolism [17]. In liver cirrhosis hepatocellular carcinoma (LC-HCC) patients, the butyrate-producing genera are found to be decreased, while the genera producing lipopolysaccharides (LPSs) increased [4]. In HBV-acute-on-chronic liver failure (HBV-ACLF) patients, alterations in circulating microbiota are also associated with multiple metabolic changes and systemic inflammation, which can affect clinical outcomes [10,20].

## 3. HBV Treatment Effect on Microbiota

Not all patients with HBV infection require or receive treatment; the effect of medication composition on the microbiota in the HBV treatment patient group has been explored. Multiple studies focused on HBV medication in relation to the microbiota. The available evidence suggests that long-term treatment (mentioned treatment options—tenofovir dipivoxil (TDF), tenofovir alafenamide (TAF), entecavir (ETV) combined with Biejiajian Pill (BJJP), ETV mono-therapy) could help restore a more balanced microbiota in HBV patients [21,25,26,27,28]. *Clinacanthus nutans* has also been used in HBV therapy, and in a murine model, *C. nutans* has been found to decrease the expression levels of HBsAg in the serum of mice [29].

## 4. HbeAg Seroconversion

Three fecal microbiota studies were identified regarding the HBeAg seroconversion-related microbial signature and prediction model for HBeAg seroconversion. HBeAg seroconversion (the loss of serum hepatitis B e antigen (HBeAg) and the development of anti-HBe antibodies) marks a transition from the immune-active (IA) phase of disease to the inactive carrier state. Results indicated that the those that reached seroconversion induced by oral antiviral therapy with tenofovir and entecavir tend to have a higher abundance of the *Firmicutes phylum* and a lower abundance of the *Bacteroidetes phylum* [30]. Interestingly, results also indicate that patients with a higher abundance of *Prevotellaceae* and genus *Sutterella* have fewer opportunities to obtain HBeAg seroconversion [30]. *Akkermansia muciniphila* has also been found to be enriched in immune-active phase patients and associated with HBeAg loss and early HBe-seroconversion [31].

However, oral microbiome compositions were not found to be significantly different in HBeAg-negative patients compared to the HCs who were vaccinated against HBV [15].

## 5. HBV DNA Load and Functional Cure

HBV DNA load is one of the factors that are taken into consideration when a decision of treatment is discussed. Research has compared high and low HBV DNA loads (>2000 IU/mL versus <2000 IU/mL) against HC. *Alloprevotella* and *Eubacterium coprostanoligenes* were found to be dominant in the low viral load group compared to the controls [14]. The gut microbiome in HBV-infected individuals with a low viral load was found to be highly diverse [14]. Serum HBV DNA has also been shown to be negatively correlated with fecal *Lactobacillus* counts [32].

In traditional Chinese medicine, tongue coating is important for clinical syndrome differentiation and drug choice; however, this method lacks clear scientific basis. An interesting relationship between tongue coating and HBV-DNA has been described in CHB patients, showing that the yellow tongue coating patients had higher HBV-DNA titers compared with the CHB white tongue coating patients (median 21,210 vs. 500, respectively), and a significantly lower level of *Bacteroidetes*, but a higher level of *Proteobacteria* at the phylum level [33]. It was also noted that the abundance of *Neisseriaceae* in the yellow tongue patients additionally positively correlated with the HBV-DNA level [33]. However, more reproducible research would be needed to draw precise conclusions about the connection between HBV DNA load and the tongue coating microbiome.

Functional cure (FC) has also been studied. Functional HBV cure is defined as undetectable HBsAg and unquantifiable serum HBV DNA for at least 24 weeks after a finite course of therapy. The gut microbiota of patients who achieved FC has been assessed and compared with that of patients with high-titer HBV DNA or low-titer HBV DNA. There was no difference found in the alpha or beta diversity of the gut microbiota between the FC group and the other groups [34]. However, the FC group presented a greater relative abundance of bacteria that produce short-chain fatty acids (SCFAs), especially butyrate, and the findings suggested that butyrate-producing bacteria contribute to FC, possibly through butyrate-mediated inhibition of HBV production [34].

## 6. ALT and AST Levels

Ten studies analyzed serum alanine aminotransferase (ALT), aspartate aminotransferase (AST) levels, and their possible connection to the microbiome; the results were heterogeneous [3,5,10,11,21,31,35,36,37,38]. Overall, the research supports a connection between different bacterial abundance and levels of ALT and AST.

Evidence suggests the following:

(1) Decrease in *Turicibacter* in HBV-LC was associated with increased AST [21];
(2) Significant decreases in *Akkermaniacaee*, *Muribaculateae*, (*Eubacterium*) *Coprostanoligenes* group, *RF39* negatively correlate with indicators of AST, ALT [5];
(3) Decreases in *Lactobacillus*, *Clostridium*, and *Bifidobacterium* were found in HBV patients with normal ALT when compared to [35] HCs; additionally, these patients were found to have more *Anaerostipes* in their gut [37];
(4) Decrease in *Lactobacillus* has been associated with higher ALT in HBV patients [36];
(5) *Desulfovibrio* and *Megasphaera* have shown positive correlations, while *Acidaminococcus* exhibits a negative correlation with high ALT levels in HBV patients [37];
(6) The pro-inflammatory bacteria (Veillonella, Escherichia-Shigella) increased counts were found to be associated with ALT and AST elevations in HBV-LC patients [30];
(7) *Prevotella*, *Blautia*, *Ruminococcus*, *Eubacterium eligens* group, *Bacteroides uniformis*, and *Ruminococcus* sp. 5_1_39BFAA have been associated with the critical biochemical indicators (ALT) and liver injury in CHB [38];
(8) *Akkermansia muciniphila* was found to be predominantly enriched in IA patients and associated with ALT flares [31];
(9) The ATL and AST levels in HBV-ACLF patients were found to be positively correlated with the abundance of *Moryella* and *Fusobacterium*, respectively [10,11].

## 7. Bile Acids in HBV

Bile acids have a function in maintaining the balance in the gut and regulating microbial growth. The specific role of bile acids (BAs) remains unclear in HBV patients; nevertheless, the virus causes hepatic inflammation and can alter bile acid metabolism. We found four studies regarding this aspect; nevertheless, they support a link with the microbiome [5,31,35,39]. Even without apparent liver injury, CHB patients have been linked to a significantly higher percentage of primary BAs and conjugated BAs [39]. Alteration of the gut microbiota and its function in bile acid homeostasis in CHB patients with different degrees of fibrosis has been explored. Analysis of gut microbiota exhibits a trend of decreased abundance in bacterial genera responsible for BA metabolism in patients with moderate/advanced fibrosis [39]. In HBV-IT patients, cholesterol-to-bile acid metabolism was found to be increased [31]. It has been proposed that *R. gnavus* facilitates HBV persistence and prolongs the IT phase, possibly through encoding bile salt hydrolase to deconjugate primary BAs, which further augments the total BAs pool [31]. In contrast, *A. muciniphila* counteracts this previously mentioned activity, possibly through the direct removal of cholesterol, which inhibits the growth and function of *R. gnavus* and promotes HBV elimination [31].

Analyzing HBV-HCC patients, the research has linked increased counts of *Acinetobacter*, *Pantoea*, *Paenibacillus*, and *Pseudomonas* with an increase in the total bile acids and triglycerides (TGs) [5].

## 8. HBV Clearance and Microbiota

In terms of microbiological factors regarding HBV clearance, research is proposing different mechanisms; for example, acute or chronic infection presentation and factors such as the age of infection, antibiotic use, cell receptors, and specific bacterial species, and nucleoside analogue therapy play a role. Research proposes probiotics as an adjunct to HBV therapy to promote HBV clearance, as probiotics have been found to accelerate serum HbsAg and HBV DNA decline in mice [40]. Evidence further points to age as being a strong factor for HBV clearance [41,42]. Research found that the sterilization of gut microbiota using antibiotics in younger mice prevented them from rapidly clearing HBV [41,43]. In murine experiments, antibiotic use can cause dysbiosis in mice and facilitate HBV persistence [27,43]. These discoveries indirectly support the connection between microbiota and HBV. Additionally, Toll-like receptors (TLRs) have been studied and could be the potential mechanism through which bacteria can influence liver inflammation. An alteration or a mutation in these receptors could lead to faster HBV clearance [41,44]. A murine study analyzed the role of Peyer’s patches (PPs) in gut microbiota-deficient mice and the HBV clearance context [45]. The authors concluded that depletion of gut microbiota impaired systemic adaptive immune responses, resulting in a delayed HBV antigen clearance [45]. In regard to specific bacterial involvement, *Ruminococcus gnavus* and *Akkermansia muciniphila* may play opposite roles in HBV infection [31]. *A. muciniphila* metabolites were found to benefit the elimination of HBV [31]. Evidence indicates that butyrate-producing bacteria as a group can contribute to FC in HBeAg-negative patients with CHB through butyrate-mediated inhibition of HBV production [34]. We additionally identified two experiments regarding nucleoside analogs (NAs) and HBV clearance [25,46]. When patients were characterized by their dominant genus, the *Bacteroides* dominant group was associated with virological undetectability; however, the *Blautia* dominant group was associated with fibrosis evolution during NAs therapy in chronic HBV [46]. Increased *Escherichia coli* relative abundance was found in on-treatment patients with advanced fibrosis despite HBV DNA undetectability [46]. However, when describing ETV’s effect on murine microbiota, it was observed that the abundance of *Akkermansia* was negatively correlated with HBV DNA load both in serum and the liver [25]. Antibiotics have been studied in regard to this topic in four studies [44,47,48,49]. Data presented that antibiotics could have a beneficial effect in certain cases where possibly translocated gut-derived microbial products promote liver injury [44]. However, further studies propose more negative effects of antibiotic use in HBV conditions. Antibiotic treatment has been demonstrated to be insufficient in altering levels of serological HBV antigens, intrahepatic HBV RNA transcripts, and HBc protein (mouse model), and has been found to contribute to HBsAg increase after breaking of IT [47]. Mice, when treated with broad-spectrum antibiotics, demonstrated gut microbiota depletion, which was further associated with impairment of colon epithelial integrity and live commensal gut microbiota translocation to the liver [48]. Furthermore, normal mice have been found to exhibit complete clearance of HBV within 6 weeks post-hydrodynamic injection (HDI) of HBV-containing plasmid, whereas multiple antibiotic treated mice have been documented to have lost this capacity, showing high serum levels of HBsAg without hepatitis B surface antibodies (anti-HBs) [49]. The authors of the research also concluded that each antibiotic alone could not significantly influence HBV clearance compared to the antibiotic combination, suggesting that global commensal microbial load is critical for promoting HBV clearance [49].

In regard to this topic, occult HBV infection (OBI) has also been studied. It is defined as the presence of HBV DNA in the liver (with detectable (usually very low HBV DNA < 200 IU/mL) or undetectable HBV DNA in the serum) of individuals testing HBsAg negative by currently available assays [50]. Interestingly, research found that the abundance of *Faecalibacterium* was significantly reduced in samples from OBI blood donors compared with those from healthy blood donors [51]. Compared with samples from HBV carriers, the samples from OBI blood donors had a significantly increased abundance of *Subdoligranulum*, which might stimulate immune activation, thus inhibiting HBV replication and contributing to the formation of occult infection [51].

## 9. HBV Patient Microbiome

**Figure 1:**
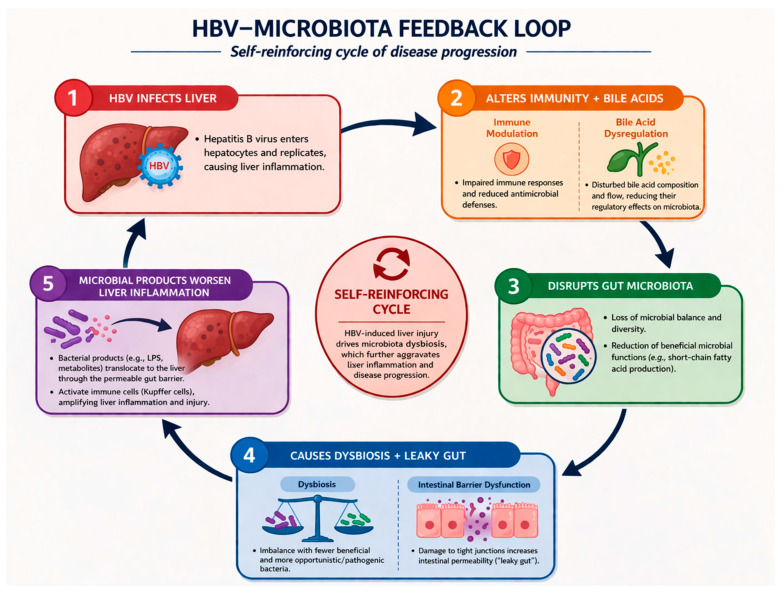
HBV-microbiota feedback loop.

Multiple studies have analyzed, but have not yet reached a consensus in terms of a unified HBV characteristic microbiome signature. HBV potentially alters the microbiome and causes a self-reinforcing cycle of disease progression through bile acid metabolism, impairment of host immunity, chronic systemic and hepatic inflammation, and intestinal barrier disruption (Figure 1). The researchers conclude that community structures and diversity of the gut microbiota in CHB patients become more unbalanced and decrease [5,9,52,53]. The available literature suggests HBV as the driver behind the changes in microbiota [13,16,52]; however, microbiota could also potentially affect HBV clinical progression [54,55]. Interestingly, *Bacteroidetes* and *Firmicutes* were found to be decreased from healthy subjects, hepatitis B virus-infected, and CHB to HBV-LC patients, while *Proteobacteria* and *Actinobacteria* showed an increasing distribution [52]. Research additionally links some bacteria to an increased risk of developing CHB, such as an increase in *Family XIII*, genus *Eggerthella* group, genus *Eubacterium ventriosum* group, genus *Holdemania*, and *Ruminococcus gauvreauii* group [54]. Additionally, ethanol-producing *Enterocloster bolteae* was found to be enriched in CHB, which could also promote chronic inflammation [55]. Bacteria, however, believed to have a protective effect against CHB are noted—*Family XIII* AD3011 group, genus *Prevotella* 7, for instance [54].

In HBV patients overall, sources propose an abundance of the following:

(1) *Actinomycetales*, *Micromonosporaceae*, *Cryomorphaceae*, and *Prevotellaceae* [6].
(2) *Fusobacteria*, *Veillonella*, and *Haemophilus* [52].
(3) *Actinomyces*, *Clostridium sensu stricto*, *unclassified Lachnospiraceae*, *Megamonas* [16].
(4) *Clostridia*, such as *Clostridium perfringens*, *Clostridium sporogenes*, *Enterocloster aldenensis*, *Enterocloster bolteae*, *Enterocloster clostridioformis*, and *Clostridium innocuum* [55].
(5) *Bacteroidetes* [23].
(6) *Lachnospiraceae* [9]. (*Haemophilus* and *Roseburia*) [9].
(7) At the genus level, *Dialister*, *Eubacterium hallii* group, *Halomonas*, *Collinsella*, *Sphingomonas*, *Xanthomonadaceae* unclassified, and *Rhizobiaceae* unclassified [26].
(8) *Fusobacterium*, *Filifactor*, *Eubacterium*, *Parvimonas*, and *Treponema*, *phylum* level *Firmicutes* and *Spirochaetes* (saliva) [53].
(9) *Streptococcus*, *Blautia*, *Veillonella*, *Fusobacterium*, and *Akkermansia* [12].
(10) *Rikenellaceae*, *Lachnospiraceae*, *Barnesiellaceae*, *Megamonas*, *Alistipes*, *Erysipelatoclostridium* [24]. *Alloprevotella*, *Paraprevotella*, and *Hungatella* [14].
(11) *Fecalibacterium*, *Streptococcus*, *Sutterella*, *Lachnospiraceae* ND-3007, *Ruminiclostridium* 9, *Lachnospiraceae* UCG-010 [21].
(12) *Lautropia*, *Abiotrophia*, and *Veillonella* were enriched in HBV patients’ saliva [56].

After our analysis, the most dominant phyla of bacteria in the HBV patient microbiome overall seem to be *Bacillota* (*Firmicutes*), followed by *Bacteroidota*—this discovery also corresponds to “normal” gut microbiome structure; however, it is followed by *Pseudomonadota* (*Proteobacteria*), which is associated with inflammation, dysbiosis, and contains pathobionts.

In sources, the HBV-LC group had a higher abundance of the following:

(1) *Veillonella*, *Megasphaera*, *Dialister*, *Atopobium*, and *Prevotella* [57].
(2) *Blautia_A* (e.g., *B. wexlerae*, *B. massiliensis*, and *B. obeum*), Dorea (e.g., *D. longicatena* and *D. formicigenerans*), *Streptococcus*, *Erysipelatoclostridium* [58].
(3) *Bacteroidetes* [23].
(4) The pro-inflammatory bacteria (*Veillonella*, *Escherichia-Shigella*) [3].
(5) *Pasteurellaceae*, *Enterobacteriaceae*, *Campylobacteraceae*, *Streptococcaceae*, and *Leptotrichiaceae* [52].
(6) *Lactobacillus* [9].
(7) *Proteobacteria* (patients with LC accompanied by ascites) [59].
(8) *Phyllobacterium*, *Sphingomonas*, *Enterococcus*, *Erysipelatoclostridium*, and *Romboutsia* [4].
(9) *Escherichia-Shigella*, *Streptococcus* [2].
(10) *Proteobacteria*, *Actinobacteria*, and *Fusobacteria*. The family level of microbiota, such as *Bacteroidaceae*, *Enterobacteriaceae*, *Sutterellaceae*, *Bifidobacteriaceae*, *Streptococcaee*, and *Pasteurellacaee* [5].
(11) *Enterobacteriaceae* (additionally, the abundance of *Steptococcus* and *Ruminococcus* was higher in patients with decompensated cirrhosis than in those with compensated cirrhosis) [60].
(12) *Bacteroidia*, *Streptococcaceae*, *Streptococcus*, *Veillonella*, *Bacteroidales*, *Lactobacillales*, *Pasteurellales*, and *Veillonella parvula* [18].
(13) *Fusobacterium*, *Catonella*, *Eubacterium*, *Filifactor*, and *Yersinia* (saliva) [53].
(14) *Veillonella* [61].
(15) *Proteobacteria* [17].
(16) *Treponema*, *Selenomonas*, and *Oribacterium* (HBV-LC patients’ saliva) [56].

Interestingly, evidence also suggests that the HBV-related cirrhosis patient microbiome can be set apart from that of alcoholic cirrhosis [62].

The HBV-HCC group had a higher abundance of the following:

(1) *Bacteroidetes* [23].
(2) *Holdemanell* [9].
(3) *Escherichia-Shigella*, *Streptococcus* [2].
(4) *Sarcina* [4].
(5) *Proteobacteria*, *Actinobacteria*, and *Fusobacteria*. The family level of microbiota, such as *Bacteroidaceae*, *Enterobacteriaceae*, *Sutterellaceae*, *Bifidobacteriaceae*, *Streptococcaee*, and *Pasteurellacaee* [5].
(6) *Blautia*, *Escherichia-Shigella*, *Bifidobacterium*, *Klebsiella*, *Parasutterella*, *E. hallii group*, *Collinsella*, *Erysipelotrichaceae UCG-003*, *Lactococcus* [21].
(7) *Haemophilus*, *Porphyromonas*, and *Filifactor* (in saliva) [56].

HBV-acute liver cirrhosis (HBV-ACLF) patients were found to be enriched in:

(1) *Moraxellaceae*, *Sulfurovum*, *Comamonas*, and *Burkholderiaceae*. Additionally, the relative abundance of *Prevotellaceae* independently predicted 28-day mortality [20].
(2) *Veillonella*, *Streptococcus*, *Enterococcus*, and *Klebsiella* (phylum level *Firmicutes*, *Proteobacteria*, *Actinobacteria*) [10].
(3) *Proteobacteria*, *Firmicutes*, *Bacteroidetes*, *Actinobacteria* (genus level *Bacteroides*, *Veillonella*, and *Streptococcus*) [11].
(4) *Parabacteroides distasonis* [20].

Interestingly, a high abundance of *Enterococcus* was associated with progression, while that of *Faecalibacterium* was associated with regression of HBV-ACLF [8].

## 10. Gut Microbiome and HBV-Related HCC

HBV infection is a major global cause of HBV-related hepatocellular carcinoma (HCC) [63]. There have been multiple studies proposing a connection between HBV-related HCC and the microbiome through different mechanisms.

The gut microbiome is a complex entity in close proximity to the liver, and the gut–liver axis has long been recognized. Gut microbiota produces excreted substances, such as SCFA, LPS, enzymes, vitamins, and other byproducts of bacteria, viruses, protozoa, fungi, and archaea, that travel to the liver through portal circulation and potentially can induce or suppress inflammation, carcinogenesis, which, combined with HBV presence in liver tissue, could help promote HCC development.

## 11. Gut Microbiota Signatures in Different Aspects and Disease Stages of HBV-HCC

Multiple studies in humans and murine models have looked at the microbiome and HBV connection to HCC risk, recurrence, and different HBV-HCC patient microbiome signatures according to age, stage of the disease, and other factors included.

We identified two studies that were conducted to identify a gut microbiota signature in differentiating between viral-related HCC (viral-HCC) and non-hepatitis B- and non-hepatitis C-related HCC (NBNC-HCC) [64,65]. The evidence supports that dysbiosis is connected to hepatocarcinogenesis and that the gut microbiota composition is significantly altered in different etiological factors of HCC. A microbiota-based signature has been found to distinguish between viral-HCC and NBNC-HCC [64,65]. Research has tried to predict the risk of HCC in HBV patients; the evidence proposes that gut microbes are important factors between HBV and HCC through potential mediating and moderating effects [66], and a similar link has been documented in mice [67]. Hepatectomies, for instance, were significantly associated with a gut microbial imbalance in HBV-HCC patients, and a significant elevation of *Klebsiella* abundance was observed in post-hepatectomy liver failure (PHLF) patients [68].

Multiple microbiota biomarkers are considered promising with the aim of providing clinicians useful tools against HCC—predicting occurrence [6,66,69] and recurrence of HCC [70], aid treatment strategy [71], predict liver failure after surgical intervention [71], moderate treatment response [72], serving as potential treatment targets [66,73], additional early diagnostic tools [69], and evidence links certain microbial genera to the size of HCC [4].

Evidence shows that the gut microbes *Bacteroidia* and *Bacteroidales* have been demonstrated to exert mediating effects between HBV and HCC; the moderating effects vary across *Bacilli*, *Lactobacillales*, *Erysipelotrichaceae*, *Actinomyces*, and *Roseburia*, and in general, HBV-gut microbe models could be established to predict HCC [66]. However, when noting HCC recurrence, *Dialister*, *Veillonella*, the *Eubacterium coprostanoligenes* group, *Lactobacillus* genus, the *Streptococcus pneumoniae*, and *Bifidobacterium faecale* species were associated with and may help predict early recurrence of HCC [70]. Gut microbial diversity has been found significantly different in studies in HC, patients with HBV and LC, and HCC compared to each other, and these microbiome characteristics could serve as potential diagnostic and treatment targets [4]. The evidence further indicates that the levels of inflammation, oxidative stress, and adiponectin are closely correlated with the progression of CHB to LC and HCC [6]. Biomarkers, such as *Enterococcus*, *Limnobacter*, and *Phyllobacterium*, could be used for precision diagnosis in HCC patients [4]. Research has also taken into consideration the changes in microbiota induced by HCC immunotherapy, specifically nivolumab [72]. Several taxa specific to therapeutic response have been designated as follows: *Dialister pneumosintes*, *Escherichia coli*, *Lactobacillus reuteri*, *Streptococcus mutans*, *Enterococcus faecium*, *Streptococcus gordonii*, *Veillonella atypica*, *Granulicatella* sp., and *Trichuris trichiura* for the non-responders to therapy; *Citrobacter freundii*, *Azospirillum* sp., and *Enterococcus durans* for the responders [72]. Interestingly, a skewed *Firmicutes/Bacteroidetes* ratio and a low *Prevotella/Bacteroides* ratio could serve as predictive markers of non-response, whereas the presence of *Akkermansia* species predicted a good response [72]. Additionally, early antibiotic exposure might have a negative effect on HCC disease outcome in patients treated with immune checkpoint inhibitors, but also in those treated with tyrosine kinase inhibitors and placebo [74]. However, more research is definitely needed in this field.

## 12. Bacteria Have Been Recognized as Potential Pathobionts or Protective Influences

Gut bacteria are capable of having positive (Table 2) or negative (Table 1) effects on health; this section highlights and presents in tables some of the identified specific bacteria and their potential proposed effects on liver health.

We conclude that most identified sources that demonstrate a protective microbiological effect promote the bacteria that are part of the probiotic group as protective substances in HBV, HBV-LC and HCC groups (Table 2).

## 13. Bile Acids and Other Markers’ Role in HBV-HCC

We identified two studies that focused on bile acids in the gut–liver axis and HCC [83,84]. It was acknowledged that changes in the tumor immune microenvironment caused by the gut microbiota via serum bile acids may be important factors associated with tumor burden and adverse clinical outcomes [84]. There is evidence that higher concentrations of bile acids, specifically conjugated primary bile acids, are associated with increased HCC risk; however, the data do not support the hypothesis that higher levels of secondary bile acids increase liver cancer risk [83].

The role of other potential markers has additionally been explored, such as serum zonulin level (a gut protein that increases the permeability of tight junctions) [85]. It was found to be significantly increased both in HBV-LC and in HBV-HCC, and correlated with the advanced stage of LC and HCC [85]. Additionally, a combination of zonulin and AFP granted a significant benefit to diagnostic accuracy in differentiating LC from HCC [85]. Lastly, IL-2 was found to be highly associated with the whole gut bacterial communities of HCC and LC groups; it could be a potential marker for the future [3].

## 14. Intratumoral Microbiome

Emerging evidence supports a high prevalence of cancer type-specific microbiota residing within tumor tissues [2,86,87,88,89]. The intratumoral microbiome has been reported to regulate the development and progression of cancers [86]. The intratumoral microbiota may contribute to the promotion of the initiation and progression of cancers by DNA mutations, activating carcinogenic pathways, promoting chronic inflammation, the complement system, and initiating metastasis [90]. Studies have researched the intratumoral microbiome, its signature [87], possible prognosis prediction [87], early detection [2], and connection with immune cell infiltration in the tumor tissue. Research has aimed to characterize intratumoral microbial heterogeneity (IMH) and establish microbiome-based molecular subtyping of HBV-HCC to elucidate the correlation between IMH and HCC tumorigenesis [86]. Microbiome-based molecular subtyping demonstrated IMH of HBV-HCC that correlates with a disparity in clinical-pathologic features and tumor microenvironment (TME) [86]. In regard to microbial biomarkers in tumor and non-tumor samples, evidence has emerged that microbial biomarkers represented by *Streptococcus* displayed a constantly increasing trend during the disease transition [2]. However, *Streptococcus* is not the only bacterium associated with HBV-HCC, and the presence of several dominant microbiota species has been confirmed in hepatic tumor and non-tumor tissues, and this discovery could possibly aid in the early detection of HCC [2]. In viral hepatitis patients (HBV and hepatitis C), research has linked *Bacteroidetes*, *Firmicutes*, and *Proteobacteria* as the dominant phyla associated with HCC (genus level unclassified *Bacteroides genus*, *Romboutsia*, *Lachnospiraceae*, and *Ruminococcus gnavus*) [89]. Furthermore, in HBV-HCC, the intra-tumoral microbiota has been positively associated with increased tumor-infiltrating CD8+ T lymphocytes and two subtypes of myeloid-derived suppressor cells (MDSCs) [88]. This finding additionally indicates an inhibitory role of microbial species in antitumor immunity and the contribution to the liver tumor microenvironment [88].

## 15. Interesting Discoveries and Related Themes to HBV Microbiome

There are publications exploring beyond the previously discussed themes, which look at specific topics in relation to microbiota and HBV.

Two studies focus on the microbiota’s link to vaccination. Interestingly, *Bifidobacterium* abundance in early infancy was found to be linked to an increase in the protective efficacy of the HBV (and other) vaccine, possibly through enhanced immunologic memory [91]. However, prebiotics (specifically chicory long-chain inulin, a soluble dietary fiber extracted from chicory root (*Cichorium intybus*)) have been found to induce changes in microbiota composition, although this has not been proven to translate into an improved immune response to the HBV vaccine [92].

HBV patient microbiota in association with other coexisting conditions has been researched as well. Some examples are HBV and type 2 diabetes mellitus [93,94], sleep disturbances, hepatic encephalopathy [95], chemotherapy [96], liver transplantation [97], patients after transjugular intrahepatic portosystemic shunt (TIPS) placement [98], and patients with cirrhotic portal hypertension after undergoing a splenectomy and pericardial devascularization (SPD) [99].

In regard to HBV and steatotic liver disease, there has been identified a reduced risk of developing dyslipidemia and non-alcoholic fatty liver disease in HBV patients, which has been possibly linked to microbiota [14]. However, murine research, for instance, has found that apolipoprotein H-negative mice with persistent HBV replication display steatohepatitis and gut microbiota dysbiosis more often [100], a conclusion that underlines a potentially significant difference between human and animal research.

Researchers have also tried to analyze the predominant intestinal microbiome of asymptomatic adult carriers of HBV (as controls without any antibiotics) and liver transplantation recipients [97]. The results demonstrated that the predominant intestinal microbial diversity decreased during the perioperative period, and postoperative fecal profiles showed an increase in *Bacteroides*, *Firmicutes*, and antibiotic use could switch the balance towards opportunistic pathogens [97].

Evidence has also demonstrated a difference in HBV-LC patients who have diabetes [94]. *Proteobacteria*, *Streptococcus*, *Escherichia-Shigella*, and *Lactobacillus* were found to be increased, while the abundance of *Bacteroidota*, *Bacteroides*, *Prevotella*, *Faecalibacterium*, and *Lachnospira* decreased in the LC-diabetes mellitus (LCDM) patients compared with the HC [94].

Bacterial diversity has been researched and found to be decreased not only in HBV patients alone but also in patients with HBV coexisting with NAFLD and T2DM [93]. There was a significantly lower abundance of bacteria of *Faecalibacterium*, *Gemmiger*, and *Clostridium XIVA* genera, but a higher abundance of *Megamonas* and *Phascolarctobacterium* genera in the HBV+NAFD+T2DM group [93]. Therefore, the bacterial signatures could potentially be the “drivers” for the comorbidities; however, it is also possible that these and other comorbidities further shape the microbiome, since the distinct gut microbiome profile associated with HBV infection has a role in lipid metabolism and glucose metabolism in patients with coexistent NAFLD and T2DM [93].

Alterations in the gut microbiome and intestinal permeability in CHB patients with cirrhotic portal hypertension after undergoing a splenectomy and a pericardial devascularization (SPD) have also been investigated [99]. Patients had microbiome analysis before and after surgery (12 months later), and researchers documented that the microbiome diversity increased, and the bacterial composition came to a level similar to that of the HC [99]. This discovery promotes the theory that the microbiome could be successfully manipulated and reversed through surgical procedures that affect the gut–liver axis.

In HBV-LC patients, sleep disturbances and different degrees of hepatic encephalopathy (HE) are common issues. Interestingly, a study aimed to evaluate the association between sleep disturbances and altered gut microbiota in patients with minimal hepatic encephalopathy (MHE) caused by HBV-LC [95], and a similar study focused on HBV-LC HE patient microbiome differences compared to HBV-LC patients without HE [18]. The patient’s microbiome was found to have decreased diversity, and specific bacteria, both *S. salivarius* and *Veillonella,* were found to be associated with sleep disturbances in patients with MHE caused by HBV-LC [95], and in HBV-LC HE patients, *Pasteurellales*, *Pasteurellaceae*, *Haemophilus*, and *Selenomonas* were found to be significantly increased bacterial genera compared to other groups [18].

In the context of chemotherapy, a murine study aimed to identify the mechanisms of Paclitaxel (PTX)-induced HBV reactivation [96]. The study revealed that PTX directly causes immunosuppression and HBV replication stimulation, and PTX induced dysbiosis of gut microbiota, which could additionally promote HBV reactivation [96].

Lastly, gut microbiome alterations in patients with HBV-related portal hypertension (PH) after transjugular intrahepatic portosystemic shunt (TIPS) placement have been studied [98]. After TIPS placement, the following results were observed: (1) the abundance of *Haemophilus* and *Eggerthella* increased, whereas that of *Anaerostipes*, *Dialister*, *Butyricicoccus*, and *Oscillospira* declined in the HE group; (2) the richness of *Eggerthella*, *Streptococcus*, and *Bilophila* increased, whereas that of *Roseburia* and *Ruminococcus* decreased in the non-HE group; and (3) members from the pathogenic genus *Morganella* appeared in the HE group but not in the non-HE group [98]. Further, it has been concluded that intestinal microbiota-related synergism may predict the risk of HE following TIPS placement in patients with HBV-related PH [98].

## 16. Fungal Microbiota in HBV Patients

Fungal microbiota has also been studied; we identified two publications on this topic.

Evidence proposes that as the disease progresses, the fungal microbiome expands [101], possibly relating to antibacterial agent use and overall dysbiosis progression. Additionally, it was found that there was a higher richness of fungal species in patients with HBV-LC than in patients with CHB, and the latter was higher than that in HBV carriers and HC [101]. There has been little difference found in enteric fungal diversity between HBV carriers and HC [101].

A total of 16 different fungal species were identified in HBV-LF patients in a study, which, on the contrary, found that HC “exhibited a slightly higher level of gut fungal richness compared to HBV-LF patients” [58]. Fungi such as *Malassezia* spp. (e.g., *M. japonica* and *M. sympodialis*), *Candida* spp. (e.g., *C. parapsilosis*), and *Mucor circinelloides* were more abundant in HBV-LF patients, while *Mucor irregularis*, *Phialophora verrucosa*, *Hortaea werneckii*, *and Aspergillus fumigatus* were found to be decreased [58].

## 17. Parasitic Microbiota in HBV Patients

The parasitic microbiome in HBV has not been widely studied; however, one study has researched the parasitic oral microbiome in these patients. A study aimed to evaluate a potential link between the colonization of gingival crevices by *Entamoeba gingivalis* and HBV infection among gum disease [102]. A statistically highly significant correlation was found between the detection of *E. gingivalis* in saliva/gingival scrapings and gum disease in HBV patients compared to HC [102]. The presence of the amoeba was not related to the degree of gum disease, but to the HBV infection diagnosis [102].

## 18. Virome and HBV Patients

In terms of virome research in association with HBV, there were four studies that we identified [58,103,104,105]. The evidence proposed plasma virome in HBV patients was primarily composed of members of the *Anelloviridae* [103,104,105], followed by *Flaviviridae*, and *Hepadnaviridae* (HBV) families [104]. Research on whether the virome differs in HBV stages and patients is contradictory; there is evidence that the gut virome of HBV-LF patients is altered and enriched by *Siphoviridae*, *Myoviridae*, and *Podoviridae* viruses compared to HC [58]; however, there is research that proposes that the virome structure and dynamics do not correlate with the different stages of chronic HBV infection, nor with the administration of antiviral therapy [104].

Interestingly, in HBV liver transplant (LT) patients, the initiation of immunosuppression was found to induce a bloom of the *Anelloviridae* that can dominate the post-liver transplant plasma (post-LT) virome [105].

## 19. HBV and Fecal Microbiota Transplantation

Fecal microbiota transplantation (FMT) has shown promising results in severe alcoholic hepatitis [106] and other non-liver-associated diseases, such as recurrent *Clostridium difficile* colitis [107]. Data on FMT in HBV are limited; however, we found FMT studies in HBV infection susceptibility, outcome, HE, hepatic myelopathy, and a possible connection of microbiota, HBV, and dyslipidemia.

There was a small study done on the role of FMT in HBeAg-positive CHB in terms of its effect on HBeAg, HBsAg, and HBV DNA [108]. The results showed that in the FMT arm, 16.7% of patients had HBeAg clearance in comparison to none in the control-antiviral therapy arm; however, none of the patients in either arm had HBsAg loss, and FMT was tolerated well [108]. Research in mice has shown that reconstitution of the gut microbiota by FMT can alter the susceptibility to HBV infection and disease outcome in mice [109], and a study on dyslipidemia using HBV-infected/free human FMT on two groups of mice noticed that altered gut microbiota accompanied by HBV infection was associated with a robust increase in alpha diversity and butyrate producers, which resulted in a reduced level of triglycerides after FMT in the recipient mice group [110]. Research shows that liver function and clinical symptoms can be improved with FMT in HBV cirrhosis patients after transjugular intrahepatic portosystemic shunt (TIPS); additionally, the number of HE attacks can be decreased significantly [111]. In HBV-associated hepatic myelopathy, FMT can improve patient condition and muscle strength [112]. Interestingly, proalbuminemia has been found to significantly increase after FMT in CHB patients, combined with a significant decrease in the HBsAg [24]. Research also found that besides live bacteria, microRNAs (miRNAs) might also play a role in the development of HE, and transferring feces and fecal miRNAs from patients with HE to the recipient mice can aggravate thioacetamide-induced HE [113]. Even though evidence in available studies is leaning towards a modulatory, safe and positive effect on patient health in terms of FMT, more research is necessary to conclude a certain benefit and safety profile for FMT in HBV patients.

## 20. Discussion

Multiple studies have tried to identify the HBV characteristic microbiome; however, more precise information is needed. The issue could come from the fact that HBV patients have different disease forms; patients may have other factors influencing the microbiota, for example, excessive alcohol use, diet choices, steatohepatitis, drug-induced liver injury, comorbidities, surgical manipulations, and patients might be receiving HBV and other disease-targeted medications. Multiple human and murine studies have been included and reviewed in this PubMed literature review. As demonstrated, research directions are diverse, often conclusions include bacteria at different ranks of taxonomy, which makes robust, accurate data analysis far more challenging and almost impossible. In terms of HBV clearance, HBeAg seroconversion, metabolic changes, influence of HBV and other medications on microbiota, more research is needed to draw significant conclusions about the possible ways the microbiome could affect these subjects. Probiotics and FMT could potentially be positive influences on HBV patient health; however, they theoretically come with their individual risks and considerations, especially in immunosuppressed patients. In general, microbiome research is promising; however, more large-scale controlled studies are needed to conclude certain benefits.

## Figures and Tables

**Table 1:** Bacteria That Are Considered to Be a Risk Factor for HBV-HCC.

Bacteria That Are Considered to Be a Risk Factor for HBV-HCC	
*Cyanobacteria*	Taxa produce microcystin—hepatotoxic tumor promoter [75]
*Enterocloster species* (specifically, *Enterocloster bolteae*)	Produces ethanol [55]
*Helicobacter hepaticus*	Considered to generate a detrimental immune microenvironment by IFN-γ/p-STAT1 axis, which can promote the tumorigenesis of HBV via recruiting innate lymphoid cells [76]
*E. coli* abundance	The profile of gut microbiota associated with the presence of HCC in cirrhotic patients is characterized by increased fecal counts of *E. coli* [77]
Klebsiella	Acts on 13 amino acid-related pathways, especially significantly observed in the branched-chain amino acid (BCAA) metabolic pathway [68]

**Table 2:** Bacteria That Are Considered to Have Potentially Protective Effects in HBV.

Bacteria That Are Considered to Have Potentially Protective Effects in HBV	
*Lactobacillus brevis SR52-2*, and *Lactobacillus delbrueckii Q80*	Contribute to an improvement in gastrointestinal health and possess anti-HBV properties—strains have exhibited the capability of inhibiting the expression of HBeAg and HbsAg in research) [78]
*Lactobacillus paracasei* N1115	Supplement has demonstrated a significant increase in intestinal microbial diversity, modulation of the intestinal microbiota, improvement in liver function, and reduction in inflammatory factor levels [79]
*Bifidobacterium longum* and *Enterococcus hirae*	Possible valuable antitumor effects through CD8+ reactivation [80]
Unspecified probiotics	Retrospective study included 1267 patients with HBV-LC treated with entecavir or tenofovir and found that the incidence of HCC was significantly lower in the probiotic user than in the nonuser group [81]
*Clostridium butyricum* combined with *Bifidobacterium infantis*	In the treatment of minimal hepatic encephalopathy (MHE) in patients with HBV-LC, improved cognition and reduced venous ammonia [82]
*Alphaproteobacteria*, genus *Family XIII* AD3011 group, *Prevotella* 7	Found to exhibit a protective effect against CHB [54]
*Bifidobacterium longum*, *Lactobacillus acidophilus*, and *Enterococcus faecalis*	Demonstrated potential to accelerate serum HbsAg decline [40]

## Data Availability

The datasets generated during and/or analyzed during the current study are available in the PubMed repository, https://pubmed.ncbi.nlm.nih.gov/ (accessed on 19 March 2026).
